# MicroRNA-1280 Inhibits Invasion and Metastasis by Targeting ROCK1 in Bladder Cancer

**DOI:** 10.1371/journal.pone.0046743

**Published:** 2012-10-04

**Authors:** Shahana Majid, Altaf A. Dar, Sharanjot Saini, Varahram Shahryari, Sumit Arora, Mohd Saif Zaman, Inik Chang, Soichiro Yamamura, Takeshi Chiyomaru, Shinichiro Fukuhara, Yuichiro Tanaka, Guoren Deng, Z. Laura Tabatabai, Rajvir Dahiya

**Affiliations:** 1 Department of Urology, VA Medical Center and UCSF, San Francisco, California, United States of America; 2 Research Institute, California Pacific Medical Center, San Francisco, California, United States of America; University of Kentucky College of Medicine, United States of America

## Abstract

MicroRNAs (miRNAs) are non-protein-coding sequences that can function as oncogenes or tumor suppressor genes. This study documents the tumor suppressor role of miR-1280 in bladder cancer. Quantitative real-time PCR and *in situ* hybridization analyses showed that miR-1280 is significantly down-regulated in bladder cancer cell lines and tumors compared to a non-malignant cell line or normal tissue samples. To decipher the functional significance of miR-1280 in bladder cancer, we ectopically over-expressed miR-1280 in bladder cancer cell lines. Over-expression of miR-1280 had antiproliferative effects and impaired colony formation of bladder cancer cell lines. FACS (fluorescence activated cell sorting) analysis revealed that re-expression of miR-1280 in bladder cancer cells induced G2-M cell cycle arrest and apoptosis. Our results demonstrate that miR-1280 inhibited migration and invasion of bladder cancer cell lines. miR-1280 also attenuated ROCK1 and RhoC protein expression. Luciferase reporter assays demonstrated that oncogene ROCK1 is a direct target of miR-1280 in bladder cancer. This study also indicates that miR-1280 may be of diagnostic and prognostic importance in bladder cancer. For instance, ROC analysis showed that miR-1280 expression can distinguish between malignant and normal bladder cancer cases and Kaplan-Meier analysis revealed that patients with miR-1280 high expression had higher overall survival compared to those with low miR-1280 expression. In conclusion, this is the first study to document that miR-1280 functions as a tumor suppressor by targeting oncogene ROCK1 to invasion/migration and metastasis. Various compounds are currently being used as ROCK1 inhibitors; therefore restoration of tumor suppressor miR-1280 might be therapeutically useful either alone or in combination with these compounds in the treatment of bladder cancer.

## Introduction

MicroRNAs (miRNAs) are non-protein-coding sequences thought to regulate >90% of human genes [1]. Deregulation of miRNA expression has been identified in a number of cancers [2], [3], and accumulating evidence indicates that some miRNAs can function as oncogenes or tumor suppressor genes. miRNAs are expressed in a tissue-specific manner and can play important roles in cell proliferation, apoptosis, and differentiation [4], [5]. Inactivation of oncogenic miRNAs [6], [7] or restoration of tumor-suppressor miRNAs [8], [9], [10] may have great potential for cancer treatment.

**Figure 1:**
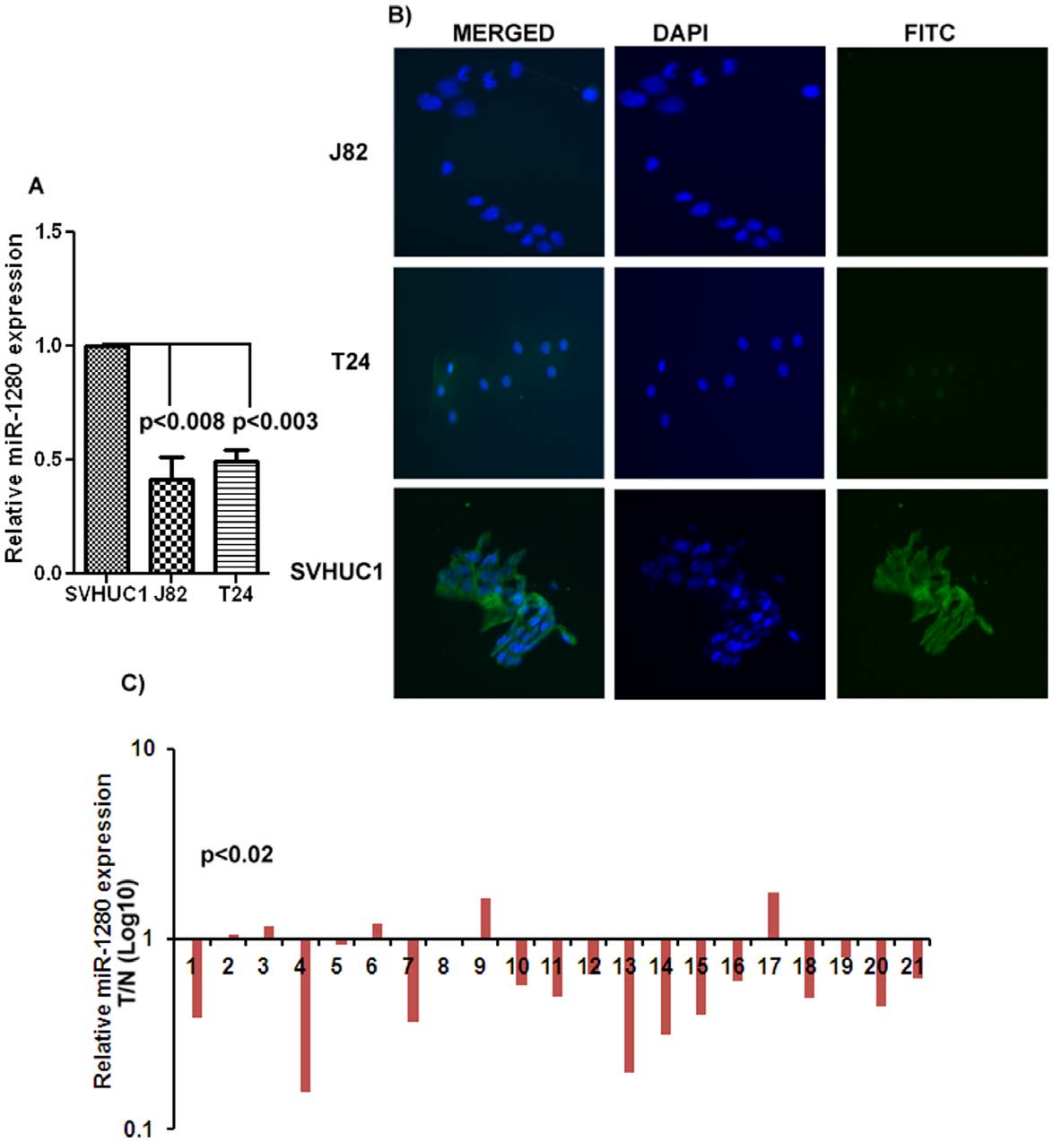
miR-1280 expression is downregulated in bladder cancer. A) Quantitative RT-PCR analysis of miR-1280 in cell lines. B) *Fluorescence In-situ* hybridization (FISH) in cell lines. C) Quantitative real time PCR analysis of mir-23b expression in matched Laser-Captured Microdissected tissue samples. (T/N- Tumor/Normal).

Alterations in cellular functions such as cell proliferation, adhesion and motility are based on the morphological changes that result from actin cytoskeleton reorganization. Rho family proteins interact with the actin cytoskeleton regulating formation of stress fibers and focal adhesions within cells. Rho-associated serine-threonine protein kinase, ROCK [11], [12], one of the best characterized downstream effectors of Rho, is activated when it selectively binds to the active GTP-bound form of Rho. Activated ROCK interacts with the actin cytoskeleton to promote stress-fiber formation and assembly of focal contacts [13]. Rearrangements of the actin cytoskeleton are involved in cancer cell migration which is central to the process of metastasis. Tang et al [14] examined the effects of ROCK1 inhibition on the activity of upstream RhoA and Rac1. ROCK1 indirectly diminishes the activity of upstream RhoA by stimulating Tiam1-induced Rac1 activity. ROCK1 provides a feedback mechanism, mediating upstream Rac1 and RhoA activity, thus reflecting the diverse effects of ROCK1 on the functional balance of small GTPases [14]. Rearranging the actin cytoskeletal proteins in response to Rho is important for the ability of tumor cells to metastasize [15]. The Rho/ROCK pathway plays role in cancer progression by regulating actin cytoskeleton reorganization and a specific ROCK inhibitor was found to suppress tumor growth and metastasis [16], [17]. In prostate carcinoma PC-3 cells, RhoA is a critical endogenous promoter of cell invasion and migration [18]. Inhibition of RhoA or its major downstream effector, ROCK1, diminishes motility of prostate carcinoma cells [18]. In bladder cancer, the Rho/ROCK pathway was reported to be involved in occurrence and progression of bladder cancer [19]. These observations suggest that the Rho/ROCK pathway may be a molecular target for prevention of cancer invasion and metastasis. Here for the first time we report on the tumor suppressor activity of microRNA-1280 (miR-1280) in bladder cancer and show that it bladder cancer migration and invasion by directly targeting ROCK1.

**Figure 2:**
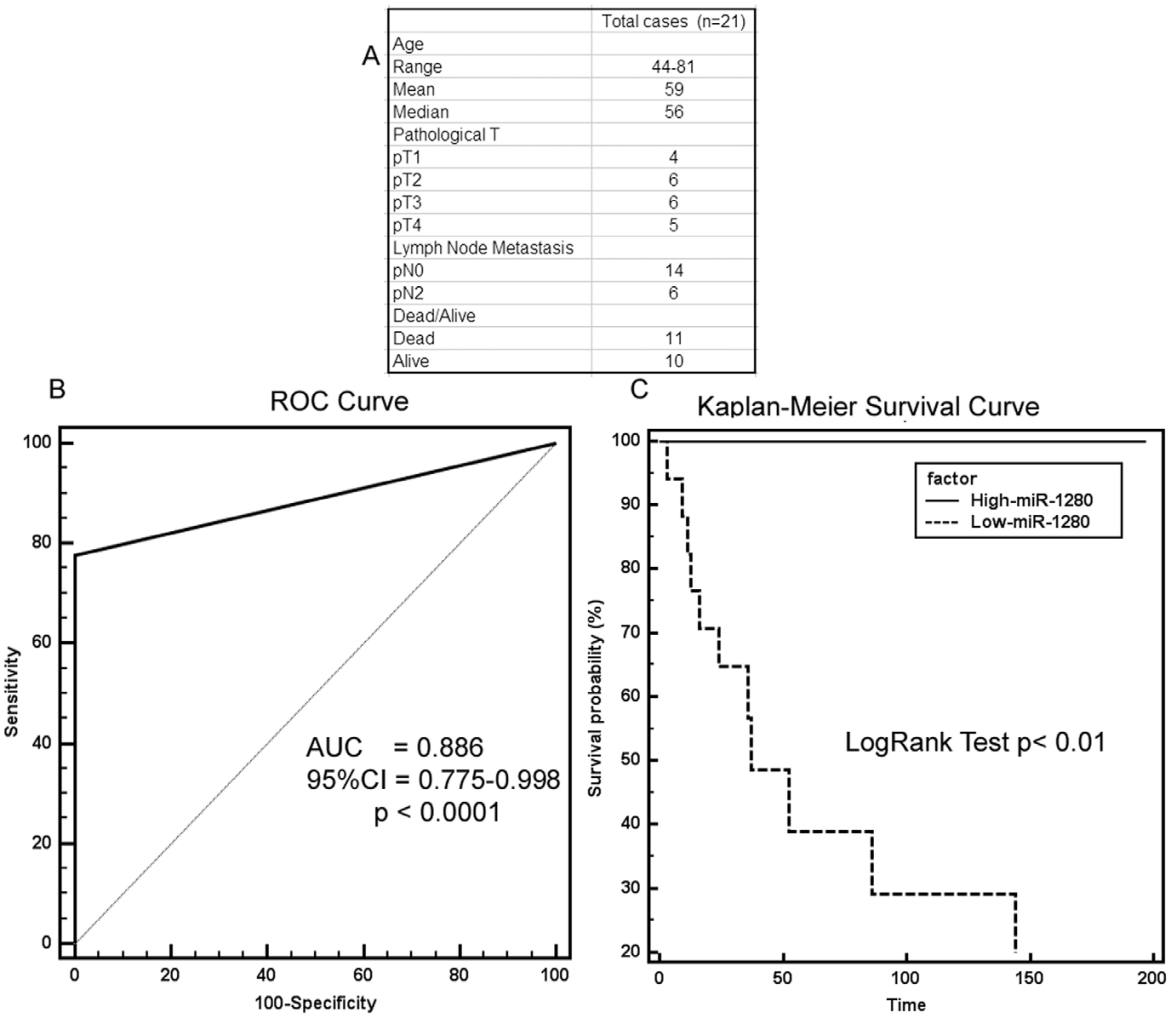
Diagnostic and prognostic significance of miR-1280 in bladder cancer. A) Clinicopathological characteristics of patient cohort. B) ROC curve analysis showing performance of miR-1280 expression to discriminate between malignant and non-malignant tissue samples. C) Kaplan-Meier analysis for overall survival based on miR-1280 expression.

## Materials and Methods

### Cell Lines and Cell Culture

SV-HUC-1, T24 and J82 cells were purchased from the American Type Culture Collection (ATCC) and grown according to ATCC protocols. SV-HUC-1 cells were cultured in F-12K Medium (ATCC) with 10% FBS. T24 cells were cultured in McCoy’s 5A medium supplemented with 10% FBS and J82 cells were cultured in Minimum Essential Media (MEM) supplemented with 10% FBS.

**Figure 3:**
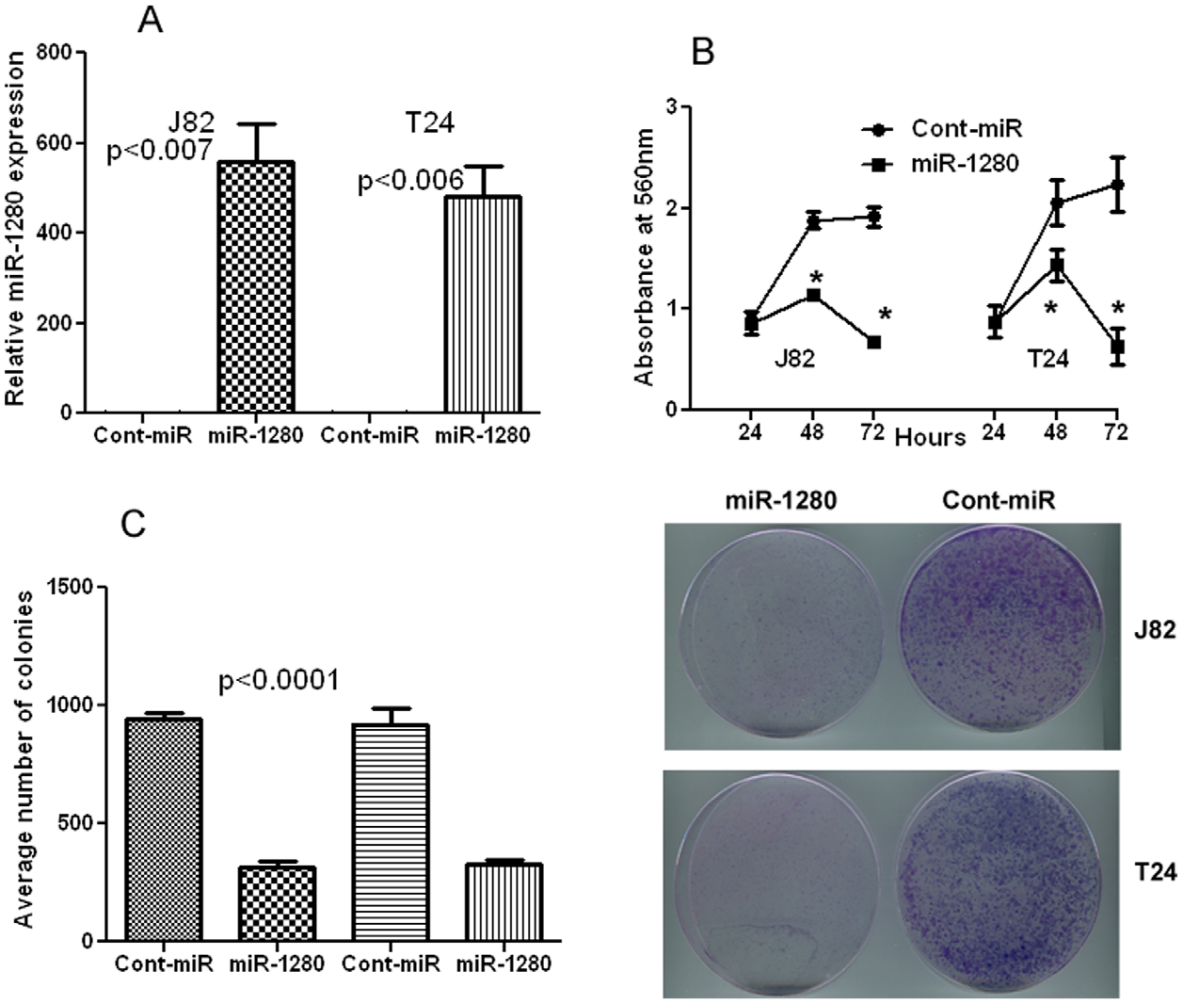
Transient transfection of miR-1280 inhibits bladder cancer cell proliferation and colony formation capability of bladder cancer cells. A) Transient transfection of miR-1280 precursor significantly increased expression of miR-1280 in bladder cancer cells. B) Proliferation of J82 and T24 cells after miR-1280 transfection was significantly reduced compared to cont-miR. C) miR-1280 over-expression significantly inhibits colony forming ability of bladder cancer cells.

### Plasmids, Precursors and Transfection

TaqMan probes and precursors for hsa-miR-1280 and negative control pre-miR were purchased from Applied Biosystems (Foster City, CA). pmir-GLO Dual-Luciferase miRNA Target Expression Vector was purchased from Promega. Lipofectamine 2000 (Invitrogen) was used for all transfections.

### RNA Extraction

miRNA and total RNA were extracted from cell lines using a miRNeasy Mini Kit and an RNeasy Mini Kit (Qiagen). miRNAs from clinical samples were extracted using laser capture microdissection techniques with a miRNeasy FFPE kit (Qiagen).

**Figure 4:**
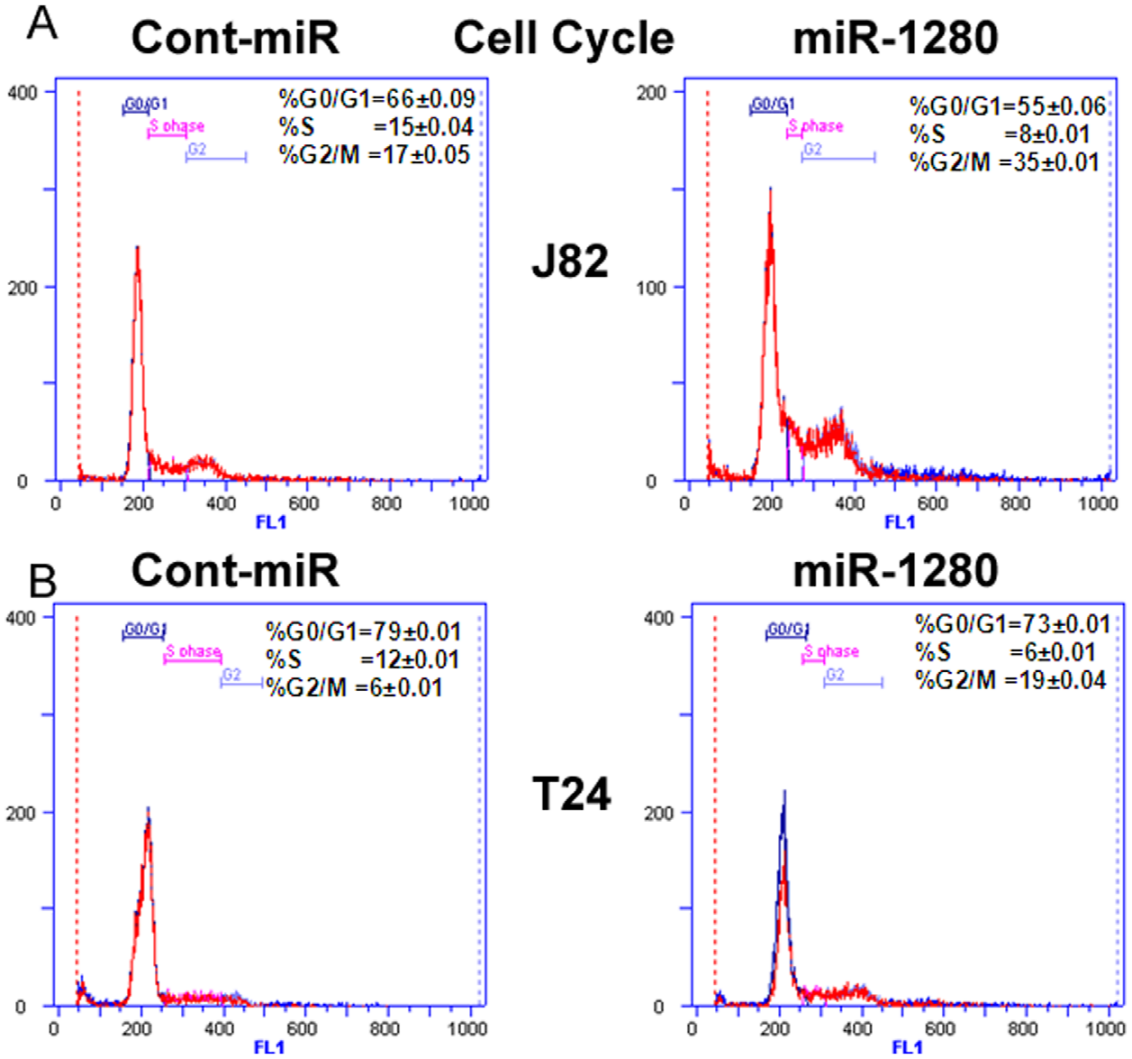
miR-1280 induces cell cycle arrest in bladder cancer cells. A-B) FACS analysis shows miR-1280 over-expression induces G2-M cell cycle arrest in J82 and T24 cells with a corresponding decrease in S-phase cells. Values are shown from triplicate experiments ±SD.

**Figure 5:**
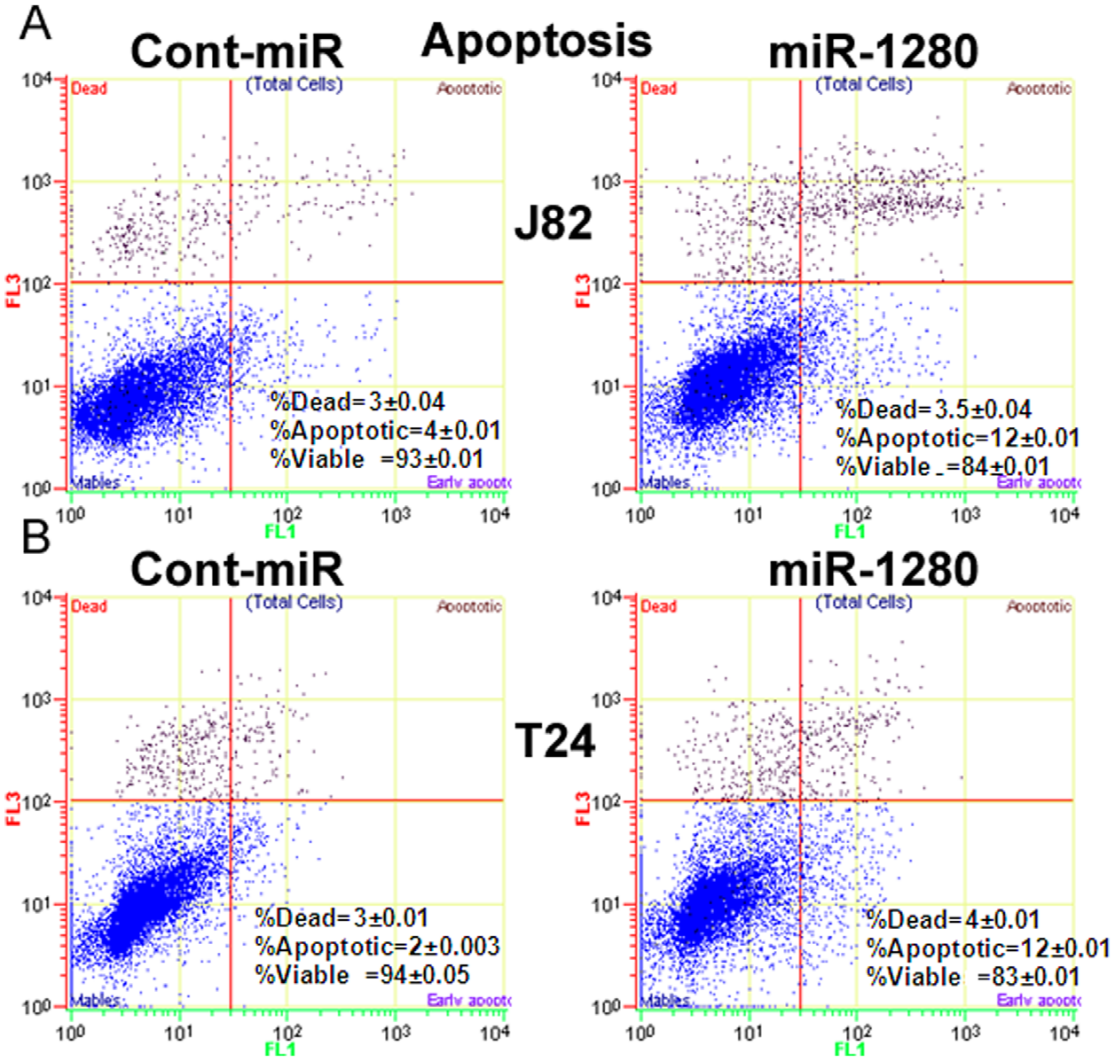
Reconstitution of miR-1280 induces apoptosis in bladder cancer cell lines. A–B) miR-1280 over-expression induces apoptosis in J82 and T24 cells with a concomitant decrease in the viable number of cells. Values shown are from triplicate experiments ±SD.

### Human Clinical Samples

Clinical samples were obtained from the San Francisco Veterans Affairs (VA) Medical Center. Written informed consent was obtained from all patients and the study was approved by the UCSF Committee on Human Research (Approval number: H9058-35751-01).

### Quantitative Real-time PCR

Mature miRNAs were assayed using the TaqMan MicroRNA Assays in accordance with the manufacturer’s instructions (Applied Biosystems). All RT reactions, including no-template controls and RT minus controls, were run in a 7500 Fast Real Time PCR System (Applied Biosystems). RNA concentrations were determined with a NanoDrop (Thermo Scientific, Rockford, IL). Samples were normalized to RNU48 (Applied Biosystems). Gene expression levels were quantified using the 7500 Fast Real Time Sequence detection system Software (Applied Biosystems). Comparative real-time PCR was performed in triplicate, including no-template controls. Relative expression was calculated using the comparative Ct.

### *In Situ* Hybridization

*In situ* hybridization was performed as described previously [20]. Briefly cell lines were stained using DIG-labeled locked nucleic acid (LNA)-based probes specific for mir-1280 following the manufacturer’s protocol (Exiqon,Inc Woburn, MA) and detected using anti-DIG-Fluorescein, Fab Fragments (Roche Applied Science, Indianapolis, IN).

**Figure 6:**
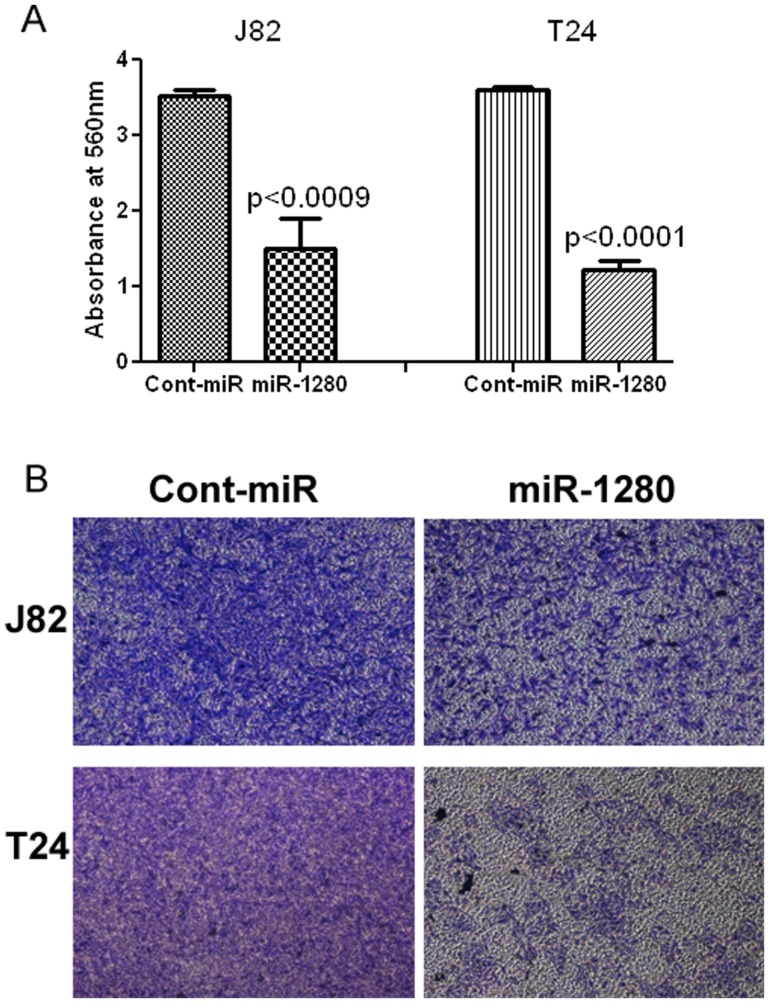
Ectopic expression of miR-1280 inhibits bladder cancer cell migration. A) Migration assays of J82 and T24 cells transfected with miR-1280. B) Representative pictures of migration assay.

**Figure 7:**
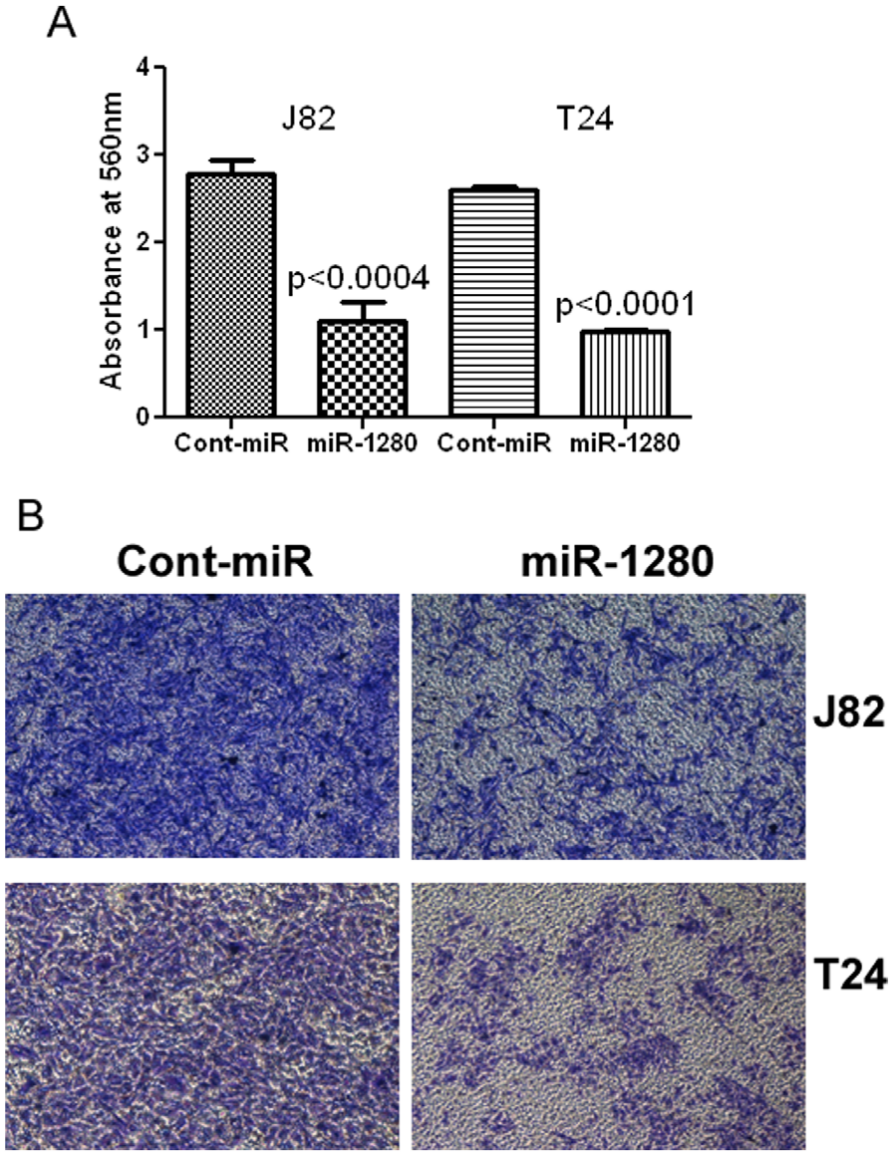
Overexpression of miR-1280 inhibits bladder cancer cell invasion. A) Invasion assay shows a significant decrease in the number of invading J82 and T24 cells transfected with miR-1280. B) Representative pictures of invasion assay.

### Cell Viability and Clonability Assay

Cell viability was determined at 24, 48 and 72 h by using the CellTiter 96 AQueous One Solution Cell Proliferation Assay kit (Promega, Madison, WI) according to the manufacturer’s protocol. Absorbance was measured at 490 nm using SpectraMAX 190 (Molecular Devices). Data are presented as the mean value for triplicate experiments compared to the negative control. For colony formation assay, cells were seeded at low density (1000 cells/plate) and allowed to grow untill visible colonies appeared. Then, cells were stained with Giemsa and colonies were counted.

### Migration and Invasion Assays

Cytoselect 24-well cell migration and invasion assay kits (Cell Biolabs, Inc) were used for migration and invasion assays according to the manufacturer’s protocol. Briefly, T24 and J82 cells transfected with Pre-miR miRNA precursor or negative control were harvested 72 hours after transfection and resuspended in serum-free Opti-MEM. Cells (10×10^4^ per 300 µl media without serum) were added to the upper chamber, and the lower chamber was filled with 500 µl of media containing 10% FBS. Cells were incubated for 16 hours at 37°C in a 5% CO2 tissue culture incubator. After 16 hours, non-migrated/non-invading cells were removed from upper side of transwel membrane filter inserts using a cotton-tipped swab. Migrated/invaded cells on the lower side were stained and the absorbance was read at 560 nm according to the manufacturer’s protocol.

**Figure 8:**
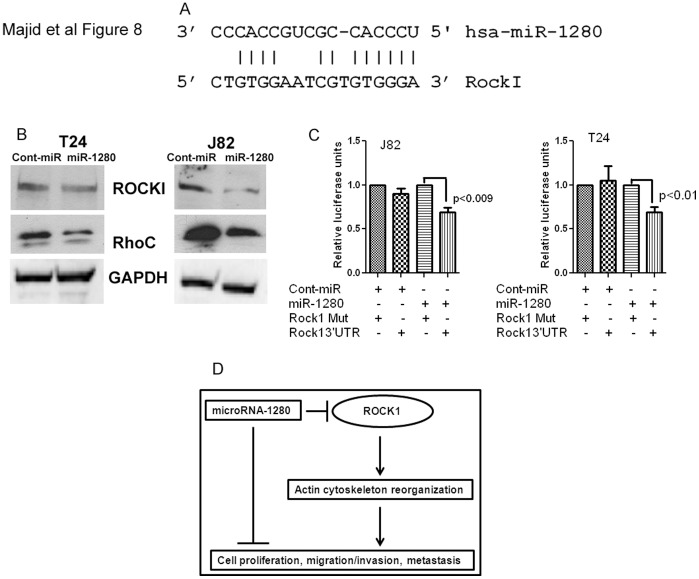
miR-1280 directly targets oncogene ROCK1. A) Complimentary miR-1280 binding sequences in the ROCK1 3′UTR. B) Western blot analysis shows that miR-1280 represses translation of oncogenes ROCK1 and RhoC. C) Luciferase assays showing decreased reporter activity after co-transfection of either the wild type or mutant Src-3′UTR with miR-1280 in J82 and T24 cells. Mut- Mutated 3′UTRROCK1 sequence. D) Schematic representation of role of miR-1280 in bladder cancer.

### Immunoblotting

Protein was isolated from 70–80% confluent plates of cultured cells using the M-PER Mammalian Protein Extraction Reagent (Pierce Biotechnology, Rockfield, IL) following the manufacturer’s directions. Protein concentrations were determined by the Bradford method. Equal amounts of protein were resolved on 4–20% sodium dodecyl sulfate (SDS) polyacrylamide gels and transferred to a nitrocellulose membrane by voltage gradient transfer. The resulting blots were blocked with 5% non-fat dry milk and probed with specific antibodies. Blots were then incubated with appropriate peroxidase-conjugated secondary antibodies and visualized using enhanced chemiluminescence (Pierce Biotechnology, Rockford, IL).

### Luciferase Reporter Assay

A pmirGLO Dual-Luciferase miRNA target expression vector was used for 3′-UTR luciferase assays (Promega, Madison, WI). The target oncogene of miRNA-1280 was selected on the basis of online microRNA target database http://www.microrna.org/microrna/home.do. The primer sequences for the wild type 3′UTR were: Forward 5′ CGCGGCCGCTAGTCTGTGGAATCGTGTGGGAT 3′ and Reverse 5′ ctagatcccacacgattccacagactagcggccgcgagct 3′. For the mutant 3′UTR, the primer sequences were: Forward 5′ CGCGGCCGCTAGTCTGTGGAATCGTTCATACT 3′ and reverse 5′ ctagagtatgaacgattccacagactagcggccgcgagct 3′. For lucifease assay, T24 and J82 cells were cotransfected with hsa-miR-1280 and pmirGLO Dual-Luciferase miRNA target expression vectors with wild-type or mutant target sequence using Lipofectamine 2000. Firefly luciferase activities were measured using the Dual Luciferase Assay (Promega, Madison, WI) 18 hr after transfection and the results were normalized with Renilla luciferase. Each reporter plasmid was transfected at least three times (on different days) and each sample was assayed in triplicate.

### Statistical Analysis

Statistical analyses were performed with GraphPad Prism 5 and MedCalc version 10.3.2. All quantified data represents an average of at least triplicate samples or as indicated. Error bars represent standard deviation of the mean. All tests were performed two tailed and *p-*values <0.05 were considered statistically significant. Receiver operating curves (ROC) were calculated to determine the potential of miR-1280 to discriminate between malignant and non-malignant samples. For disease progression, Kaplan-Meier (log-rank test) analysis was performed.

## Results

### Expression of miR-1280 in Bladder Tumors and Cancer Cell lines

Expression of miR-1280 was examined by real-time PCR in bladder cancer cell lines J82, T24 and compared to non-malignant cell line SV-HUC1. The results indicated that miR-1280 was downregulated in cancer cell lines (Figure 1A). *In-situ* hybridization also confirmed the presence of miR-1280 expression (green signal) in SV-HUC1 cells compared to cancer cell lines (Figure 1B). To examine the biological significance of miR-1280, its expression was analyzed in laser captured microdissected (LCM) human bladder tumor tissue and compared to normal matched control tissue. The expression of miR-1280 was found to be significantly downregulated in all the tumor samples compared to their matched normal samples (Figure 1C). These results indicate a putative tumor suppressor role for miR-1280 in bladder cancer.

### Diagnostic and Prognostic Significance of miR-1280 in Bladder Cancer

Clinical demographics of the patient cohort are summarized in Figure 2A. Receiver operating curve (ROC) analyses were performed to evaluate the ability of miR-1280 expression to discriminate between normal and tumor cases using tissue samples. An area under the ROC curve (AUC) of 0.886 (P<0.0001; 95% CI = 0.775 to 0.998) (Figure 2B) was obtained suggesting that miR-1280 expression can discriminate between malignant and non-malignant samples and hence can be used as a diagnostic marker for bladder cancer though additional samples may strengthen these results. To determine whether miR-1280 has any prognostic significance, we divided cases into low miR-1280 (expression T/N<0.8 fold) and high miR-1280 (expression T/N>0.8 fold) groups and performed Kaplan-Meier survival analysis. In Kaplan-Meier analysis, the high miR-1280 group had significantly higher overall survival probability compared to the low miR-1280 group (Logrank Test p<0.02) (Figure 2C). These findings suggest that miR-1280 has the potential to be a diagnostic and prognostic marker for bladder cancer.

### MicroRNA-1280 Overexpression Suppresses Bladder Cancer Cell Proliferation and Colony Formation

To determined the functional significance of miR-1280 overexpression in bladder cancer, we transfected bladder cancer cell lines J82 and T24 with miR-1280 precursors. miR-1280 was significantly overexpressed in J82 and T24 cell lines after transient transfection with miR-1280 precursor compared to the cont-miR precursor (Figure 3A). Ectopic expression of miR-1280 significantly decreased cell proliferation as compared to cells expressing cont-miR (Figure 3B). miR-1280 transfected cells also had low colony formation ability as the number of foci in miR-1280 expressing cells were decreased when compared with cont-miR transfected cells (Figure 3C). These results indicate anti-proliferative effect of miR-1280 in bladder cancer.

### miR-1280 Triggers Cell Cycle Arrest and Induces Apoptosis in Bladder Cancer Cells

FACS (fluorescence activated cell sorting) analysis revealed that re-expression of miR-1280 lead to a significant increase in the number of cells in the G2-M phase of the cell cycle (17% to 35%) while the S-phase population decreased from 15% to 8% in J82 cells (Figure 4A). Similar results were observed in T24 cells with an increase in G2-M population of cells (6% to 19%) and a decrease in S-phase population (12% to 6%), suggesting that miR-1280 triggers a G2-M arrest in miR-1280 transfected cells compared to cont-miR. FACS analysis for apoptosis was performed using Annexin-V-FITC-7-AAD dye. The percentage of total apoptotic cells (early apoptotic + apoptotic) was significantly increased (4% to 12%) in response to miR-1280 overexpression compared to cont-miR with a corresponding 10% decrease in the viable cell population in J82 cells (Figure 5A). In T24 cells, an increase (2% to 12%) in apoptotic cells was observed with miR-1280 overexpression compared to cont-miR (Figure 5B). These results indicate a tumor suppressor role for miR-1280 in bladder cancer.

### Anti-migration/Invasion Effects of miR-1280 in Bladder Cancer

Overexpression of miR-1280 had anti-migratory and anti-invasive effects on bladder cancer cell lines. Less absorbance was observed at 560 nm with miR-1280 transfected cells compared to cont-miR in the migration assay (Figure 6) and miR-1280 overexpression also significantly reduced the invasiveness of bladder cancer cells (Figure 7).

### miR-1280 Directly Targets Oncogene ROCK1

ROCK1 has been reported to be an important molecule that drives bladder cancer migration and invasion. Using an online microRNA target database we found oncogene ROCK1 as the putative target of miR-1280 with complementary 3′UTR sites for the seed sequence of miR-1280 (Figure 8A). We performed Western analysis for ROCK1 expression in miR-1280 transfected cells and found that miR-1280 attenuated the protein expression of ROCK1 compared to the cont-miR (Figure 8B). We also found a decrease in the protein levels of RohC, another oncogenic protein that is upstream of ROCK1. To check whether a direct interaction is involved between miR-1280 and its target oncogene ROCK1, we performed luciferase reporter assays. We found that co-transfection of miR-1280 along with the wild type 3′UTR of oncogene ROCK1 caused a significant decrease in luciferase units compared to controls (Figure 8C). These results suggest that miR-1280 targets oncogene ROCK1 directly.

## Discussion

Though little is known about microRNA-1280, one study has shown that miR-1280 is expressed in colon and pancreatic cancers based on expression analysis of 19 colorectal and 17 pancreatic human cancer samples [21]. Here we for the first time report that miR-1280 plays a tumor suppressor and has diagnostic and prognostic potential in bladder cancer. We also performed functional analyses to confirm the anti-tumor effects of miR-1280 and show that miR-1280 directly targets oncogene ROCK1, an important molecule in bladder cancer cell migration and invasion.

We also observed that miR-1280 is significantly downregulated in bladder cancer cell lines and tumor tissues compared to non-malignant cell line or normal tissues indicating that miR-1280 might be a tumor suppressor in bladder cancer. Previous studies have shown that microRNAs are highly tissue specific and they can act as tumor suppressor or oncogenes [5], [6]. MicroRNAs possess several features that make them attractive candidates as new prognostic biomarkers and powerful tools for the early diagnosis of cancer [22]. In this study, we found that miR-1280 was predictive of overall survival such that patients with higher miR-1280 expression had longer overall survival compared to patients with low miR-1280 expression. MicroRNA-1280 expression also distinguished malignant from normal tissues indicating the diagnostic significance of miR-1280 in bladder cancer although this needs to be confirmed in a larger cohort of tissue samples. Our functional assays revealed that miR-1280 has anti-proliferative effects, inducing cell cycle arrest and apoptosis in bladder cancer. It also showed anti-migratory and anti-invasive effect on bladder cancer cells.

Since ROCK1 is an important molecule that is involved in bladder cancer migration and invasion [19], we examined whether ROCK1 is a target of miR-1280 in bladder cancer. Overexpression of ROCK1 has been reported to occur in various cancers [14], [19] and the Rho/ROCK pathway has been found to be associated with progression of bladder cancer [19]. ROCK mediates responses in the pathway initiated by Rho, and regulates the reorganization of cytosleletal proteins such as formation of stress fibers and focal adhesions [23]. Rearranging cytoskeletal proteins is important for the ability of tumor cells to metastasize [15] and our study showed that miR-1280 attenuated expression of oncogene ROCK1. Luciferase assays also revealed direct interaction of miR-1280 and ROCK1. Therefore these results indicate that miR-1280 inhibits migration/invasion and thus metastasis of bladder cancer that is mediated through downregulation of oncogene ROCK1 (Figure 8D). We also found decreased expression of oncogene RhoC that is upstream of ROCK1. A previous study has reported that ROCK1 diminishes activity of its upstream RhoA family members indirectly [14]. By the same principal, inhibition of ROCK1 by miR-1280 may have an indirect inhibitory action on the RhoC oncogene.

In conclusion, our study is the first report to document the tumor suppressor role of miR-1280 in bladder cancer. miR-1280 directly targets oncogene ROCK1, inhibiting migration/invasion which are central to the process of metastasis. Various compounds such as Y-27632, have been found to inhibit ROCK1. Our findings indicate that restoration of tumor suppressor miR-1280 might be useful therapeutically either alone or in combination with these compounds in the treatment of bladder cancer.
